# Cause-of-death data series to monitor cause-specific mortality across time and space

**DOI:** 10.1038/s41597-026-07683-5

**Published:** 2026-06-27

**Authors:** Magali Barbieri, France Meslé, Svitlana Poniakina, Denys Dukhovnov, Domantas Jasilionis, Lisa Koerber, Dmitri Jdanov

**Affiliations:** 1 Institut national d’études démographiques, 9 cours des Humanités, CS 50004, 93322 Aubervilliers, Cedex France; 2 Department of Demography, University of California, Berkeley, 310 Social Sciences Building, Berkeley, CA 94720-2120 USA; 3 Max Planck Institute for Demographic Research, Konrad-Zuse-Straße 1, 18057 Rostock, Germany

## Abstract

Cause-specific mortality provides useful clues regarding the driving forces behind mortality trends in high income countries. Nevertheless, conventional data sources to measure cause-specific mortality trends are affected by recurring changes in the International Classification of Diseases (ICD). They also lack the degree of detail needed to construct consistent time series, at least for the periods before implementation of the 10^th^ ICD Revision. The Human Cause-of-Death Data series (HCD@HMD) is an extension of the Human Mortality Database designed to provide cause-specific mortality time series with causes of death classified according to consistent lists of causes of death, taking changes to the ICD into account. The HCD@HMD provides high-quality detailed continuous data series for three standard lists of cause-of-death categories (short, intermediate, and long) coded to the 10^th^ ICD Revision, harmonized over time and across populations. The reconstructed series are currently available for 18 countries. All HCD@HMD data series are updated periodically and include the most recent data available. The data follow the FAIR principles and they are publicly available.

## Background & Summary

The twentieth century has witnessed an extraordinary increase in the average length of life in high-income countries. In the United States for instance, life expectancy at birth rose from 47 years in 1900 to 77 years in 2000^1^. This gain, of about one year of life every three calendar years, has been generally representative of the rest of the developed world during the same period. Some notable deviations from this improving trend include Eastern European countries showing long-term stagnation or even decline in life expectancy during several decades after the mid-1960s^2,3^. In recent decades, progress has also slowed in many Western countries^4^. In particular, the United States has experienced several years of stagnation in life expectancy at birth, even before the COVID pandemic^5^. Other high-income countries, such as the United Kingdom, Germany or France, have also exhibited a slowing down in mortality decline^6^. An examination of changes in cause-specific mortality can provide crucial clues regarding the factors behind the remarkable longevity gains of the 20^th^ century and the slowing down of the past twenty years and it is essential for understanding the future of the longevity revolution. In addition, coherent and internationally comparable cause of death data are highly policy relevant because they provide detailed evidence about the potential determinants and the burden of risk factors, such as smoking or alcohol^7–9^. They thus constitute an essential tool to design and monitor effective evidence-based health interventions and policy recommendations. Nevertheless, despite the availability of large arrays of cause-specific mortality data, relying on this type of data is challenging when examining long-term trends or carrying out cross-country comparisons, due to variations in cause-of-death classifications over time and across countries.

In countries with long established vital statistics systems, cause-of-death information is routinely collected for all deaths occurring in the population, together with other important information like sex, age at death, and place of residence at the time of death. Since the end of the 19^th^ century, cause-of-death information on death certificates has been collected following international guidelines and coded to a standard scheme, the International Classification of Diseases (ICD). The ICD was originally established by health statisticians and it has been maintained since the early 20^th^ century, first by the International Statistical Institute, then by the League of Nations, and since 1948 by the World Health Organization. Cause-of-death data in countries which have adopted this codification scheme are thus comparable across populations. However, the ICD is periodically revised to account for improvement in diagnosis, public health priorities, the recognition or emergence of new pathologies – e.g., HIV-AIDS, Alzheimer’s, COVID – and to provide a greater level of details in the understanding of biological mechanisms in diseases. Though the WHO has now published an 11^th^ Revision of the ICD, it has not yet been implemented by any countries. As of 2026, cause-of-death data are coded to the 10^th^ Revision of the ICD (ICD-10) in all countries. The 10^th^ Revision of the ICD includes over 10,000 distinct conditions, compared with just over 200 in the first version of the ICD. The ICD revisions sometimes involve minor changes (e.g., ICD-7) but often entail a major reorganization of the classification (especially ICD-6 and ICD-10) and new definitions that create severe disruptions in cause-specific mortality series. For instance, the introduction of ICD-10 has led to an unprecedented increase in the level of details and the number of items included in the classification, a major reorganization of the main ICD chapters, and changes in coding rules. These disruptions hamper the understanding of long-term trends in cause-specific mortality.

When the changes involve the aggregation of several conditions into a unique category (many-to-one equivalence) or the splitting of a category into several (one-to-many), these disruptions can be easily resolved by relying on the smallest common combination of causes. However, it is often the case that new categories are created from the recombination of portions of old categories (many-to-many equivalence). In these latter situations, relationships between the various conditions are complex, making it impossible to easily track the number of deaths for one specific condition through successive revisions of the ICD. Table 1–4 provide examples of four different kinds of correspondences (one-to-one, one-to-many, many-to-one, and many-to-many) for the transition from ICD-9 to ICD-10.

**Table 1:** An example of one-on-one equivalence.

ICD-9 code	Item	ICD-10 code	Item
204.1	Chronic lymphoid leukaemia	C91.1	Chronic lymphocytic leukaemia of B-cell type

Additionally, though most countries rely on the ICD to classify and code their cause-of-death data, they have not always used the same level of detail and some countries have implemented their own classifications, such as those in the Republics of the former Soviet Union which relied on a separate scheme. These latter countries have only switched to the ICD at the end of the 20^th^ or in the early 21^st^ century when they adopted ICD-9 or ICD-10. Even when countries have eventually used the same revisions of the ICD, they have rarely done so simultaneously. Comparisons of cause-specific mortality trends over time or across countries thus create challenges that we sought to resolve through the Human Cause-of-Death project. The goal of the project was to construct historical series of detailed cause-specific mortality indicators consistent over time and across populations (i.e., taking changes in the successive ICDs into account) for as many countries with high-quality and available data as possible.

Ideally, it should be possible to reconcile successive classifications relying on bridge-coding studies. Such studies code deaths for a given year into two successive ICD schemes to allow statisticians or researchers to easily recode causes of death from the first to the second of these two schemes. However, very few countries (the United States, the United Kingdom, and France) have carried out bridge-coding studies and in all instances, except for the United States, the sample of deaths on which the studies have been carried out do not allow for more than a very broad categorization of causes of death. Even in the United States, where bridge-coding studies have been conducted for a given year on all deaths since the transition from ICD-4 to ICD-5, the information is no longer accessible except for the 1996 study, which documented the transition from ICD-9 to ICD-10^10^.

**Fig. 1:**
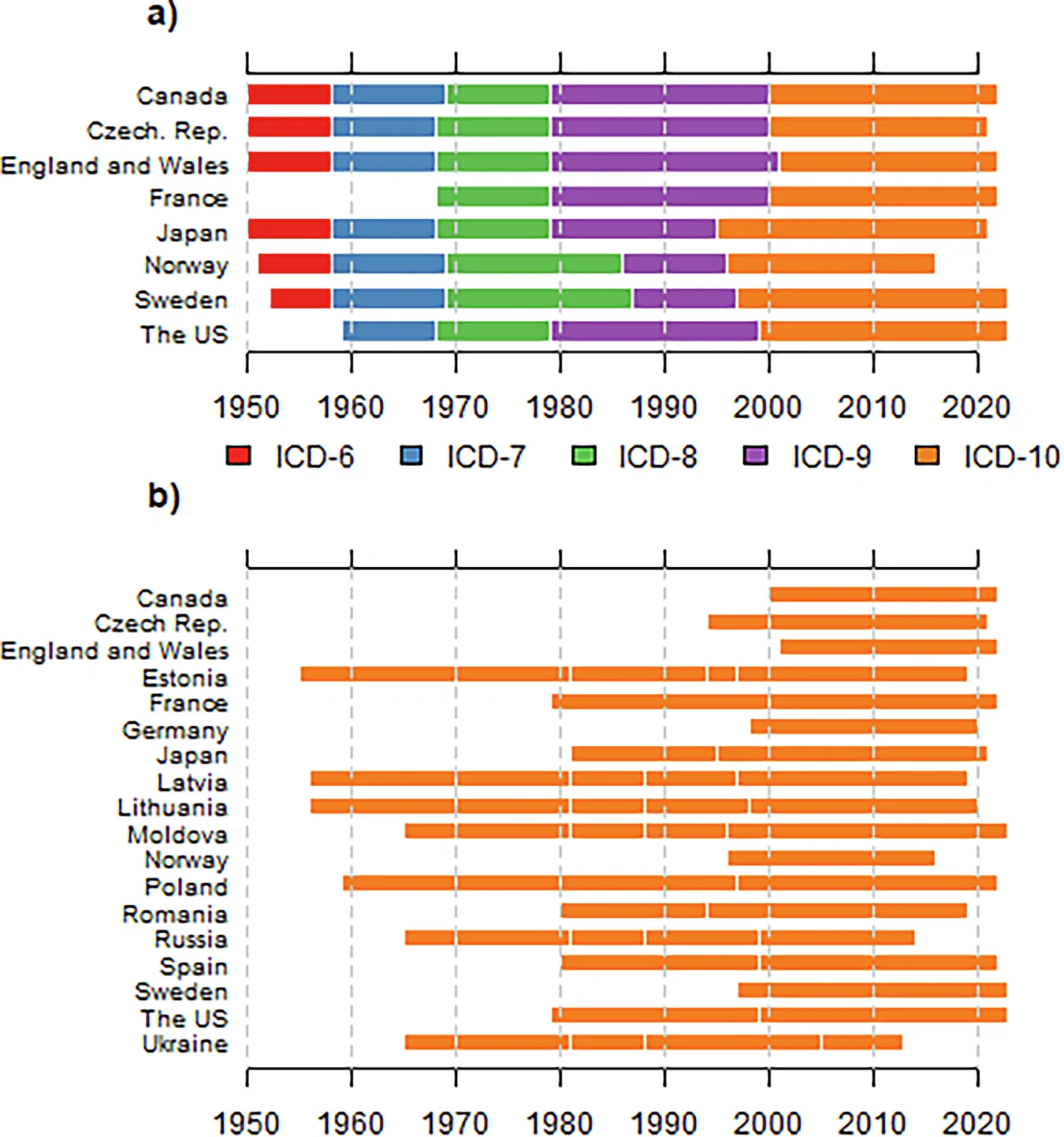
Human Cause-of-death Data series available by country and calendar year (**a**) for the series available in their original classifications (ICD-6 to ICD-10) and (**b**) for the series reconstructed into ICD-10.

To remedy this major hurdle for the understanding of the survival revolution of the 20^th^ century and the challenges of the 21^st^ century, a method has been developed by France Meslé and Jacques Vallin^11–14^. The method was originally implemented on French data for the period from 1925 to 1978^11^ and has been further developed to construct standard series of cause-specific mortality rates for 18 countries with high-quality data as part of an international project^15,16^. These data series (HCD@HMD) have been published in the Spring of 2024 within the Human Mortality Database^17^, a freely accessible database of all-cause mortality indicators for 41 high-income countries back to the 19^th^ or even 18^th^ century for some of them. However, the period covered by the cause-specific data series is more restricted and varies from country to country (Fig. 1).

There is no existing alternative to the HCD@HMD series because the WHO repository of cause-specific data^18^ only provides statistical information specific to each successive ICD, that is with the causes of death classified according to the ICD scheme in use at the time when the original data were collected. Furthermore, for periods before the implementation of ICD-10, deaths are not provided for the entire range of ICD codes but only for aggregated categories that are not consistent from one ICD period to the next. The Eurostat provides cause-specific mortality series for a consistent list of 65 broad categories but only for the recent period (since 1994) and only for countries in the European Union as well as for the four countries of the European Free Trade Association (Iceland, Liechtenstein, Norway and Switzerland) and two candidate countries (Serbia and Turkey). In contrast to the Eurostat and WHO, the HCD@HMD provides harmonized/reconstructed time series with causes of death classified according to a constant (fixed) classification, taking changes in the International Classification of Diseases into account. The time series include age-specific death rates, crude death rates, age-standardized death rates as well as the population exposures used to calculate the rates. In addition, the database is updated in a timely manner as HCD series are extended on an annual basis, when new cause-of-death data are released by the National Statistics Offices or other organizations that produce and distribute them.

## Methods

The main inputs for the HCD@HMD are country-specific de-identified tabulations of death counts by age, sex, and detailed cause of death, birth counts, and population estimates for national populations distributed by National Statistics Offices and derived from the vital statistics system and population censuses. The exact sources of raw data for each country and calendar year are listed in a country-specific reference file (see Usage Notes). The reference file lists all sources of data, organized by the type of statistical information collected (deaths, births, or population). The reference file indicates the names of the data providers, as well as the corresponding URLs for data retrieved from the Internet, and the date at which the information was obtained. In some cases, particularly for earlier periods, the data may have come from a researcher’s personal collection (e.g., digitized from a physical archive). In such cases, the name of the researcher who has provided the data to the project is indicated. The raw data, formatted as tabulations of death counts with the causes of death reported in their original classification (i.e., in the ICD Revision implemented at the time when the data was collected), are only available for countries and time periods when authorizations from the data providers have been secured (Fig. 1a).

The cause of death of interest is the underlying cause of death, that is the “the disease or injury that initiated the train of morbid events leading directly to death, or the circumstances of the accident or violence which produced the fatal injury”^19^. The HCD relies on cause-of-death data coded in either the ICD scheme or in a Soviet scheme (including the country-specific classification used by the former German Democratic Republic). The Soviet Classification was designed to provide an alternative to the ICD in countries of the former Soviet Union, where the government published statistics for some particular causes of death (cholera, plague, suicide, homicide, and occupational accidents) separately for political reasons^20^. Like the ICD, the Soviet classification was revised multiple times to adjust to public health priorities as well as to progress in diagnosis and medical science up to the 1990s, when the 9^th^ and then the 10^th^ Revisions of the ICD were progressively adopted in all the countries of the former USSR. Some former Soviet Republics (the three Baltic countries, Belarus, Moldova, and Ukraine) have used slightly modified versions of the Russian classifications, but the variations are small enough to enable direct comparisons of the cause-of-death statistics in all these countries.

The HCD@HMD provides time series of mortality indicators with causes of death grouped according to standard lists, taking changes to the ICD into account. There are three major steps to construct the indicators. First, cause-specific series of death counts by age and sex are reconstructed at the most detailed level (i.e., classified into 3- or 4-digit ICD-10 codes whatever the ICD Revision implemented at the time the death records were established). Next, deaths are aggregated into larger cause-of-death categories following the standard lists developed for the project: a long list, an intermediate list, and a short list. Last, mortality indicators are calculated for each country series as further described below.

### Reconstruction of cause-specific mortality

The first step is to re-classify into ICD-10 the causes for deaths collected under a previous ICD scheme. The key treatment here is the calculation of transition coefficients. The coefficients are country-, age- and cause-specific. There are two different approaches to the calculation of transition coefficients. As previously mentioned, the gold standard is bridge-coding, that is the dual classification of deaths simultaneously into the old and new coding schemes. When bridge-coding has been performed at a detailed level and made available by the national office in charge of processing cause-of-death data, transition coefficients can be inferred directly from the results. Transition coefficients are based on the results of bridge-coding studies whenever is possible. However, even for the transition from ICD-9 to ICD-10 in the United States, not all ICD-9 and ICD-10 codes are represented for the one calendar year (1996) when the study was conducted: 45% of all possible codes are missing from ICD-9 and 56% from ICD-10^14^. Consequently, an alternative approach must be implemented either independently, in the absence of bridge-coding, or as a complement to the bridge-coding study, to redistribute deaths from one ICD period into the categories of the following ICD period.

Additionally, some changes may occur outside the official transition to a new Revision of the ICD. These may be implemented by the World Health Organization itself (for instance with the introduction of new ICD codes, as with the addition of COVID-related conditions in 2020) or they may be country-specific, due to shifts in coding practices, changes in definitions, or updates to a given version of the classification. These additional changes are described in the HCD country-specific documentation files and when they are associated with visible disruptions in the data series, corrections are applied *a posteriori*, following the same method as for the transition from one ICD Revision to another. For each country and each transition from one ICD scheme to another, the approach involves the following steps:

1. First, two so-called “correspondence” tables are constructed. The correspondence tables list, for each item in one of two successive ICD Revisions, all the items of the other ICD Revision to which they partially or completely contribute in terms of medical content. One such table is constructed for all the items in the first of the two ICD Revisions in comparison with those in the next ICD Revision and another table is constructed for all the items in the second of the two ICD Revisions in comparison with those in the previous ICD Revision.
2. Next, the smallest possible combinations of items containing the same medical contents in both classifications are identified from the correspondence tables (see Tables 1–4 above for examples). These groups of items are called “fundamental associations”. Country specialists verify the statistical consistency over time of each association and adjust the correspondence tables, when necessary, that is when the death rate series for a given association exhibits a statistically significant disruption at the time of the ICD transition.

**Table 2:** An example of one-to-many equivalence.

ICD-9 code	Item	ICD-10 code	Item
205.0	Acute myeloid leukaemia	C92.0	Acute myeloblastic leukaemia
		C92.4	Acute promyelocytic leukaemia
		C92.5	Acute myelomonocytic leukaemia

**Table 3:** An example of many-to-one equivalence.

ICD-9 code	Item	ICD-10 code	Item
015.0	Tuberculosis of bones and joints, Vertebral column	A18.0	Tuberculosis of bones and joints
015.1	Tuberculosis of bones and joints, Hip		
015.2	Tuberculosis of bones and joints, Knee		
015.7	Tuberculosis of bones and joints, Other specified bone		
015.8	Tuberculosis of bones and joints, Other specified joint		
015.9	Tuberculosis of bones and joints, unspecified

**Table 4:** An example of many-to-many equivalence.

ICD-9 code	Item	ICD-10 code	Item
070	Viral hepatitis	B15	Acute hepatitis A
		B16	Acute hepatitis B
		B17	Other acute viral hepatitis
571.1	Acute alcoholic hepatitis	B18	Chronic viral hepatitis
		B19	Unspecified viral hepatitis
571.4	Chronic hepatitis	K73	Chronic hepatitis, not elsewhere classified
		K70.1	Alcoholic hepatitis
3. Then, transition coefficients between all the items within each of the fundamental associations are calculated and applied to redistribute deaths coded under a given ICD Revision into the items of the next ICD Revision.
4. The results derived from the medical logic of the classification rules have to be checked statistically to detect and solve any remaining breaks in the series. Such checks are carried out visually by age group and sex implementing the statistical tools developed for this purpose^21,22^.
5. Deaths of unknown ages, which typically represent a very small share of all deaths (<1%), are redistributed proportionately over each sex and cause-of-death category.
6. Deaths of ill-defined or unknown causes are redistributed for each sex and within each age group into well-defined cause-of-death categories. The share of deaths from unknown and ill-defined causes varies across countries and time periods and depends on reporting and certification practices. This complicates international comparisons of cause-specific mortality. There are different ways to deal with this challenge to improve comparability of cause-of-death statistics across populations^23–25^. In the HCD, the redistribution of deaths from ill-defined and unknown causes is carried out using various methods, depending on the time period and on the country, either proportionately within each age group and for each sex independently, or using linear relationships in cause-specific trends^26^. The specific method implemented for each transition is described in the country-specific Background and Documentation files. Such deaths typically represent a very small share of all deaths (<1%) with some exceptions, namely for Lithuania and Poland in the 1950s (with a high of 23% in Lithuania in 1956) and for more recent years in France, Japan, and Poland (around 10%–12%).
7. The last step consists in verifying the results of the reconstruction through rigorous validation. Cause-specific and age-specific mortality trends are systematically reviewed to identify any remaining inconsistencies or implausible breaks in the series.

**Fig. 2:**
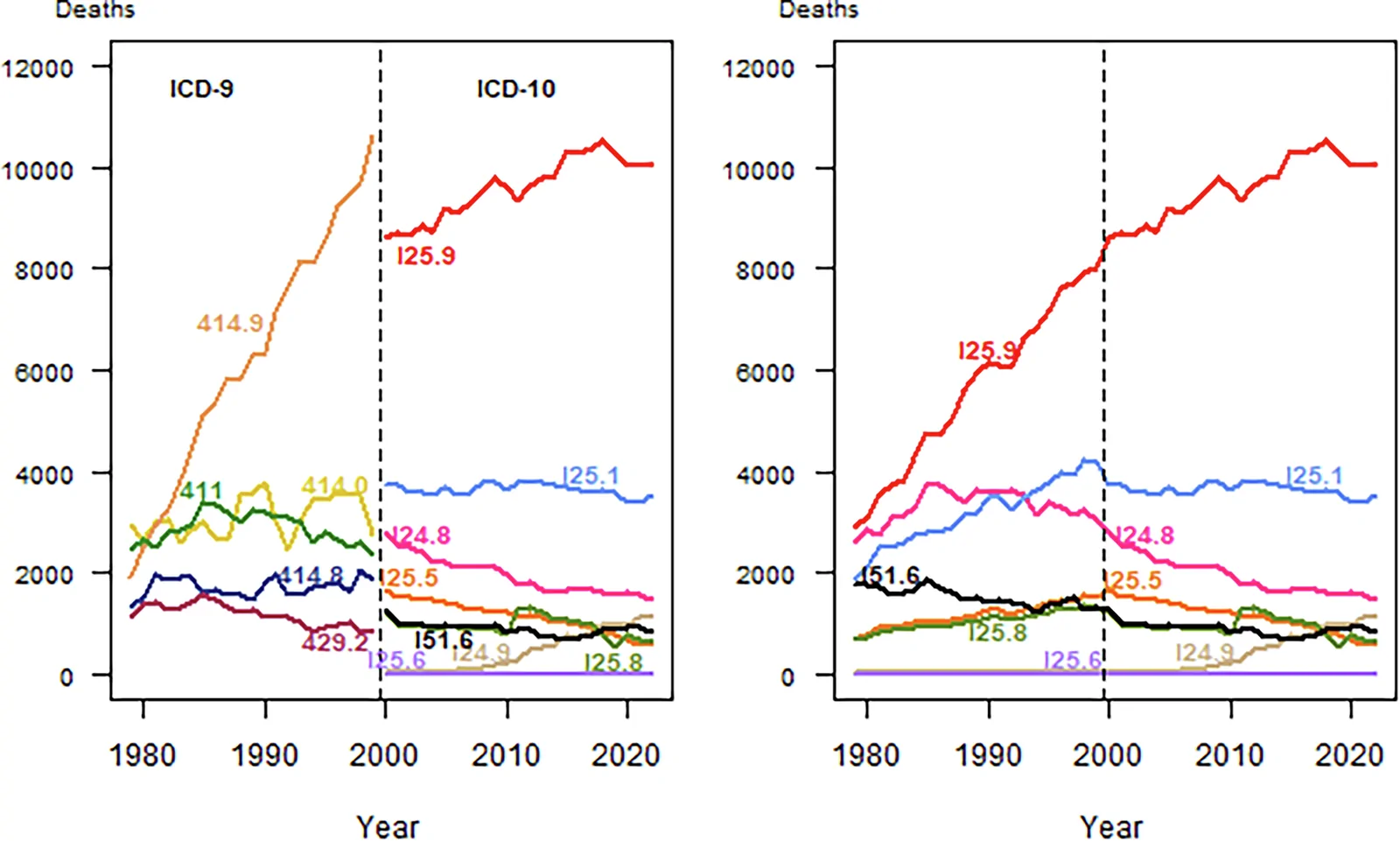
Cause-specific death counts for ischaemic heart diseases in France, from 1979 to 2023 (**a**) in their original classification and (**b**) after reclassification into ICD-10, with corresponding 3-digit ICD codes. **ICD-9 codes:** 411 Other acute and subacute forms of ischaemic heart disease. 414.0 Coronary atherosclerosis. 414.8 Other specified forms of chronic ischaemic heart disease. 414.9 Chronic ischaemic heart disease, unspecified. 429.2 Cardiovascular disease, unspecified. **ICD-10 codes:** I24.8 Other forms of acute ischaemic heart disease. I24.9 Acute ischaemic heart disease, unspecified. I25.1 Atherosclerotic heart disease. I25.5 Ischaemic cardiomyopathy. I25.6 Silent myocardial ischaemia. I25.8 Other forms of chronic ischaemic heart disease. I25.9 Chronic ischaemic heart disease, unspecified. I151.6 Cardiovascular disease, unspecified.

These steps are established as a guideline, but the work is tailored to each country independently, up to step 6 (step 7 is carried out by the project coordinator using automated tools and under supervision by the project Directors). Indeed, it is not possible to construct correspondence tables that would be applicable to all transitions in all countries or to use the transition coefficients calculated for one country to reconstruct the series for another country. This is because of the high degree of country-specific practices in filling out death certificates and coding causes of death, especially for older ICD transitions, when standardization was not as developed as it is today. Standardization has been achieved in part due to the adoption of the WHO-recommended death certificate in most countries and the automation of coding and selection of the underlying cause of death following the rules established by WHO, with an increasing degree of precision over time. All country-specific adjustments are detailed in the country-specific documentation available on the HCD website^27^. Figure 2 illustrates the results of the analysis by comparing the death counts for ischaemic heart diseases in France before and after reconstruction. This is an example of a fundamental association with many-to-many equivalences. The ICD-9 and ICD-10 codes included in the association are listed under the Figure.

### Selected causes of death

The data are first reconstructed into the most detailed list of causes of death available in each country, at either the 3- or 4-digit levels of the ICD for all countries using the WHO coding scheme. For those using Soviet classifications, the reconstruction is conducted at the most detailed level possible with the complete list of codes. Death counts by sex, age and calendar year are then aggregated into a full list of (3-digit) ICD-10 codes, and then into the HCD@HMD standard long, intermediate, and short lists of cause-of-death categories, which are nested within each other. The lists have been designed to include separate categories for the leading causes of death as well as policy-relevant categories. More information about each list is provided below, and in Table 5.

- The Full list for countries implementing the WHO classification of diseases corresponds to the 2016 revision of ICD-10 and includes 1643 items at the 3-digit level, excluding asterisk codes, post-procedural disorders and codes which cannot be used for the underlying cause of death. After redistribution of deaths from ill-defined or unknown causes to well-defined categories, the number of items is reduced to 1555. For Russia, the Baltic states, Moldova and Belarus, the Full lists are different, ranging from 237 items for Russia to 267 items for Ukraine, reduced by several items after redistribution of deaths from ill-defined or unknown causes.
- The long list is the most detailed of the three standard lists of cause-of-death categories (i.e., identical for all countries and time periods in the HCD). It has been designed in particular for bridging the Soviet classification implemented by many Eastern European countries before they adopted ICD-10 with the International Classification of Diseases proposed by WHO. The list includes 209 categories. After redistribution of deaths from ill-defined or unknown causes, the number of categories is reduced to 206.
- The intermediate list aggregates death into 58 categories. After redistribution of deaths from ill-defined or unknown causes, the number of categories is reduced to 56.
- The short list is the most abridged of the HCD@HMD lists of cause-of-death categories, aggregating deaths over the chapters or sub-chapters of the International Classification of Diseases. It includes 17 categories, or 16 without deaths from ill-defined or unknown causes.

**Table 5:** Cause-of-death classifications.

List identifier	Description	Number of items	
		including ill-defined	excluding ill-defined
full	ICD10 3-digit	1643	1555
long	CoD long list	209	206
interm	CoD intermediate list	58	56
short	CoD short list	17	16
belarus	Belarus list	266	264
ukraine	Ukraine list	271	268
baltic	Baltic and Moldovan lists	243	240
russia	Russian list	237	235

Note that for the reconstructed data series corresponding to each of the three shortlists (long, intermediate and short), we provide the mortality indicators after redistribution of deaths from ill-defined and unknown causes (as previously described). We also separately provide a series of death counts from ill-defined and unknown causes for researchers seeking more information about these.

The long, intermediate, and short lists of cause-of-death categories as well as the ill-defined and unknown cause-of-death categories in each list with all their corresponding ICD-10 codes are included into the compressed zip file with all data posted to the Harvard Dataverse^28^. All well-defined and ill-defined cause-of-death categories in the three lists are available as separate files in the “docs” directory that is created after the zip file is uncompressed.

### HCD mortality indicators

The reconstructed death counts by sex, age, and cause of death are combined with birth and population data to compute sex-, age- and cause-specific death rates, crude death rates, and age-standardized death rates. The population exposure and birth counts used to estimate the denominators of the mortality rates are taken directly from the corresponding all-cause series in the Human Mortality Database (HMD), with some exceptions. In particular, adjustments to the HMD exposures had to be carried out for a small subset of countries located in Eastern Europe, to account for differences in the definition of a live birth. These countries have been using a different threshold in terms of months of gestation and birth weight than those recommended by WHO, leading to an under-estimation of infant mortality. The countries for which the adjustment is necessary are Belarus, Estonia, Lithuania, Latvia, Moldova, Romania, Russia, and Ukraine. The adjustment consists of re-integrating both the infant death count and the birth count for those babies who should not have been excluded^29^.

The crude death rate is the simplest and most commonly-used measure of mortality. For any given population and calendar year *t*, the crude death rate for a given cause of death *c*, ${{CDR}}^{c}(t)$, is computed by simply dividing the total number of deaths from cause *c*, ${D}^{c}\left(t\right)$, by the total number of person years lived in the population over that given year (what demographers call the “population exposures”), *E*(*t*):

1 $${{CDR}}^{c}\left(t\right)=\frac{{D}^{c}\left(t\right)}{E\left(t\right)}$$

The age-specific death rates for a given cause of death *c*, ${m}^{c}(x,t)$, are calculated in each country by dividing the number of deaths from cause *c* in a given calendar year at any given age *x*, ${D}^{c}(x,t)$, by the population exposures in the same year at the corresponding age *x*, *E*(*x*, *t*). The age-specific death rates are provided for the following age groups: infants, 1–4 years and every five-year age groups thereafter up to open age intervals of 85+, 90+, 95+ and 100+ years, depending on the level of age detail for that given year in that given country:

2 $${M}^{c}(x,t)=\frac{{D}^{c}(x,t)}{E(x,t)},x=0,1,5,10,\ldots ,85+$$

The age-standardized death rates are provided to facilitate comparisons across countries and over time^30^. The calculation of an age-standardized death rate supposes the selection of a reference, or standard, population age structure. Because most of the countries in the HCD are European countries, we have chosen to compute age-standardized death rates relying on the two standard structures proposed by the Eurostat, i.e., the 1976 and the 2013 standards (Table 6).

**Table 6:** 1976 and 2013 European Population Standards.

Age group	1976 standard	2013 standard
0	1,600	1,000
1–4	6,400	4,000
5–9	7,000	5,500
10–14	7,000	5,500
15–19	7,000	5,500
20–24	7,000	6,000
25–29	7,000	6,000
30–34	7,000	6,500
35–39	7,000	7,000
40–44	7,000	7,000
45–49	7,000	7,000
50–54	7,000	7,000
55–59	6,000	6,500
60–64	5,000	6,000
65–69	4,000	5,500
70–74	3,000	5,000
75–79	2,000	4,000
80–84	1,000	2,500
85–89	600	1,500
90–94	270	800
95–99	95	145
100+	35	55
Total	100,000	100,000

***Source***: European Commission, 2013^33^.

Age-standardized death rates are presented for all ages combined and for broad age groups, that is at 0–14 years, 15–39 years, 40–64 years, and 65 years and above. In any given population and for any calendar year, the age-standardized death rate for a given cause of death *c* and the age group *x to x* + *n*, ${SD}{R}^{c}([x,x+n),t)$, is calculated as follows:

3 $${SD}{R}^{c}([x,x+n),t)=\frac{\sum _{x\le y < x+n}{M}^{c}\left(y,t\right){E}^{{ESP}}(y)}{{\sum }_{x\le y < x+n}{E}^{{ESP}}(y)}$$

where ${E}^{{ESP}}(y)$ represents the population exposure at age *y* in the standard age structure.

## Data Records

The HCD provides access to a series of country-specific web pages from which the users can access all country-specific data, metadata, and background information. A snapshot (as of January 20, 2026) of the compressed file with all country statistics combined is available through the Harvard Dataverse repository^28^. Permanent storage and updated data series are available from the HCD portal web page at the HMD^17^. Two types of data series are made available, appearing on separate panels on the HCD country pages: the series are either harmonized into ICD-10 or in their original classification, as further described below.

### Reconstructed mortality data series

Reconstructed mortality data series are available for 18 countries (Fig. 1a). The series range from 22 years for Germany (1998–2020) to 64 years for Estonia (1955–2019) and Lithuania (1956–2020). Separate data files are provided for each of the following indicators: death counts, age-specific death rates, crude death rates (CDR), and standardized death rates (SDR). The reconstructed series are provided for the three lists of aggregated causes of death previously described (short, intermediate, and long) and, for most countries, for a full list of ICD codes that varies from country to country. In addition, a second panel on the country pages provides access to the death counts reconstructed into the ill-defined and unknown categories of ICD-10 separately, when we are authorized to distribute the raw data.

The files are named according to the following convention: the first three digits correspond to the country ISO-3 code (e.g., USA for the United States); the following letters identify the type of data (*d* for the death counts, *m* for the age-specific death rates, *cdr* for the crude death rates, and *sdr* for the age-standardized death rates); the next portion of the name identifies the list of cause-of-death categories (full, long, interm[ediate], and short); for the death counts with ill-defined or unknown causes, the list identifier includes the mention “id” (i.e., idlong, idinterm, idshort), while the extension _idr indicates that deaths of ill-defined or unknown cause have been redistributed. So, for instance, the filename USA_d_long_idr.csv is used for the death counts classified into the long list of cause-of-death categories after redistribution of deaths from ill-defined and unknown causes for the United States.

### Original mortality data series

For reproducibility purposes, the data are also provided in the International Classification of Disease versions in which they have been originally coded, though only for countries which National Statistics Offices have authorized us to do so. The data in their original classifications, that is with deaths classified according to the ICD Revision implemented at the time when the deaths were reported, include only two series of mortality indicators: death counts and death rates by age, sex, and cause of death. A major difference between the reconstructed and unreconstructed data series is that deaths from ill-defined or unknown causes, which are redistributed in the reconstructed series, remain in separate categories in the original mortality data series. The two data series are provided in the same format as the reconstructed series though the death counts in their original classifications are provided only for the short and intermediate lists.

Original data series are available for only 8 HCD countries (Fig. 1a). The original input data (the unreconstructed data series) are not available for all other countries because of restriction in our data user agreements related to confidentiality concerns by the National Statistics Offices. Among all countries for which we have produced reconstructed data series, we have only obtained the authorization to reproduce the original information for three (i.e., France, Japan, and the United States). For another five countries (Canada, England and Wales, Norway and Sweden), we publish our estimates for the period of ICD-10 implementation only (we have not completed the reconstruction to bridge to previous revisions of the ICD) but we are in the process of reconstructing the full historical data series.

### Birth and population data series

As described as part of the Methods section, cause-specific mortality rates are calculated using birth counts (for infant mortality) and population exposures from the core HMD^17^, with some adjustments for Eastern European countries. To avoid any confusion, the exact birth and population data series used for the calculation of the rates are reproduced in the bottom panel of each HCD country page. In most countries, these are exact replicates of the HMD series. For Belarus, Estonia, Lithuania, Latvia, Russia, and Ukraine, as previously explained, some adjustments to the infant mortality rates have been implemented in the HCD@HMD but not (yet) for the core HMD. Also note that cause-of-death series are provided for two countries, Moldova and Romania, that are not yet included in the core HMD collection. Nevertheless, the population data used in the HCD@HMD for these two countries were processed following the HMD methods protocol.

### Format of the data files

The same structure is used for all the files containing the *death counts* and the *age-specific death rates*. The first line of each file includes the variable names. Each file contains 32 variables (columns). The first six columns refer to identifying variables: country, year, sex, list of causes (full, long, intermediate, or short), age group format, and cause of death category. Column 7 contains death counts or rates for all ages combined. Columns 8–32 contain deaths or rates by age group. Death counts are provided with up to 2 decimals because of the proportionate redistribution of deaths from unknown ages and unknown or ill-defined causes of death. Death rates are given per 1,000,000 population and published as integers.

For the *crude death rates* (CDRs) and the *age-standardized death rates* (SDRs) files, the first six columns of the data files are identical to the first six columns of the death files. The following columns present the CDR or SDR values, respectively. While the CDR file contains only one indicator variable (‘cdr’), the SDR file provides SDRs calculated using the two versions of the European Standard Population (Table 6).

The *population* exposure file contains 30 variables. The first four columns refer to identifying variables (country, year, sex, and age group format); column 5, to the population exposures for all ages combined; and columns 6–30, to population exposures by age group.

The *birth* file is provided with 3 identifying variables (country, year, and sex) and one variable with the actual count of live births.

All data files are comma-separated files with the extension “.csv”. A dot is used as decimal separator. Each record is unique: one line represents a unique combination of country, year, sex, and cause, which cannot be repeated in the same file. Missing values are coded as dots (“.”).

## Technical Validation

The HCD series are limited to populations included in the HMD with the (temporary) exception noted above. To be included in the HMD, a country must have complete coverage of the population by the vital statistics system and the information reported on births, deaths, and populations must be of high quality. Before deciding whether to include a new country or updated data series for an existing HMD country, extensive checks are performed to assess reliability. It follows that the HCD series are only available for countries with virtually or fully complete coverage of vital statistics and meeting strict formal criterions regarding the quality of the original cause-of-death data.

In addition to country-specific knowledge based on the HMD experience, data quality indicators specifically designed for this project are computed to check the consistency of HCD data series, both at a time when a country is considered for inclusion into the HCD and at each update. A set of standard checks has been developed to ensure that the cause-of-death series are of the highest quality. To facilitate calculation, these checks are grouped into three types: diagnostic checks, consistency checks, and graphical checks. They are described in detail in the protocol that serves as a guide for quality checks. The verifications are carried out by the database administrators using data provided by the HCD country specialists or uploaded from WHO (for updates) as input for the database and they must be approved by the project’s Directors before publication on the database website.

Death counts are checked at the most detailed level for which they are provided, i.e., typically at the 3- or 4-digit level, or some other country-specific exhaustive list of items as may be the case for the former Soviet republics. Eventually, the 4-digit list is aggregated into a 3-digit list, and data are published at the 3-digit level for the full list of cause-of-death categories.

### Diagnostic checks

This kind of checks verifies that the ICD-codes for which the input death counts are categorized are plausible. In particular, the ICD-codes must only include categories corresponding to underlying causes of death and they must exclude any morbidity-specific, asterisk or post-procedural codes, which, according to WHO guidelines, must never be used for the underlying cause of death. Since the classification as defined by WHO is constantly evolving, some obsolete codes may appear from one update to the next and we carry out these verifications each time the series are extended with new data. The reference classification for the database is the ICD-10 list for the 2016 revision.

Another type of checks involves verifying that male-specific causes only apply to males (e.g., no prostate cancer for females) and female-specific causes only apply to females (e.g., no ovarian cancer or conditions specific to female reproduction and maternal mortality for males); that causes specific to infants (e.g., infections specific to the perinatal period) do not apply to older adults and, inversely, that causes specific to older adults (e.g., senility or senile dementia) do not apply to young children; and that ICD codes corresponding to diseases introduced recently into the ICD have not been used (erroneously) in previous years, such as HIV-AIDS (introduced into the ICD in 1987), SARS (in 2003) and COVID-19 (in 2020).

### Consistency checks

Consistency checks include both internal and external checks. Internal checks verify the internal consistency of the data. For instance, we make sure that the sums of deaths by age, sex and cause matches the total counts; we compare the overall counts in the raw data to the total in the processed counts; we check the plausibility of the sex ratios in the death rates. External checks verify the consistency between the cause-specific input death counts provided for the HCD to the all-cause counts in the HMD.

### Graphical checks

**Fig. 3:**
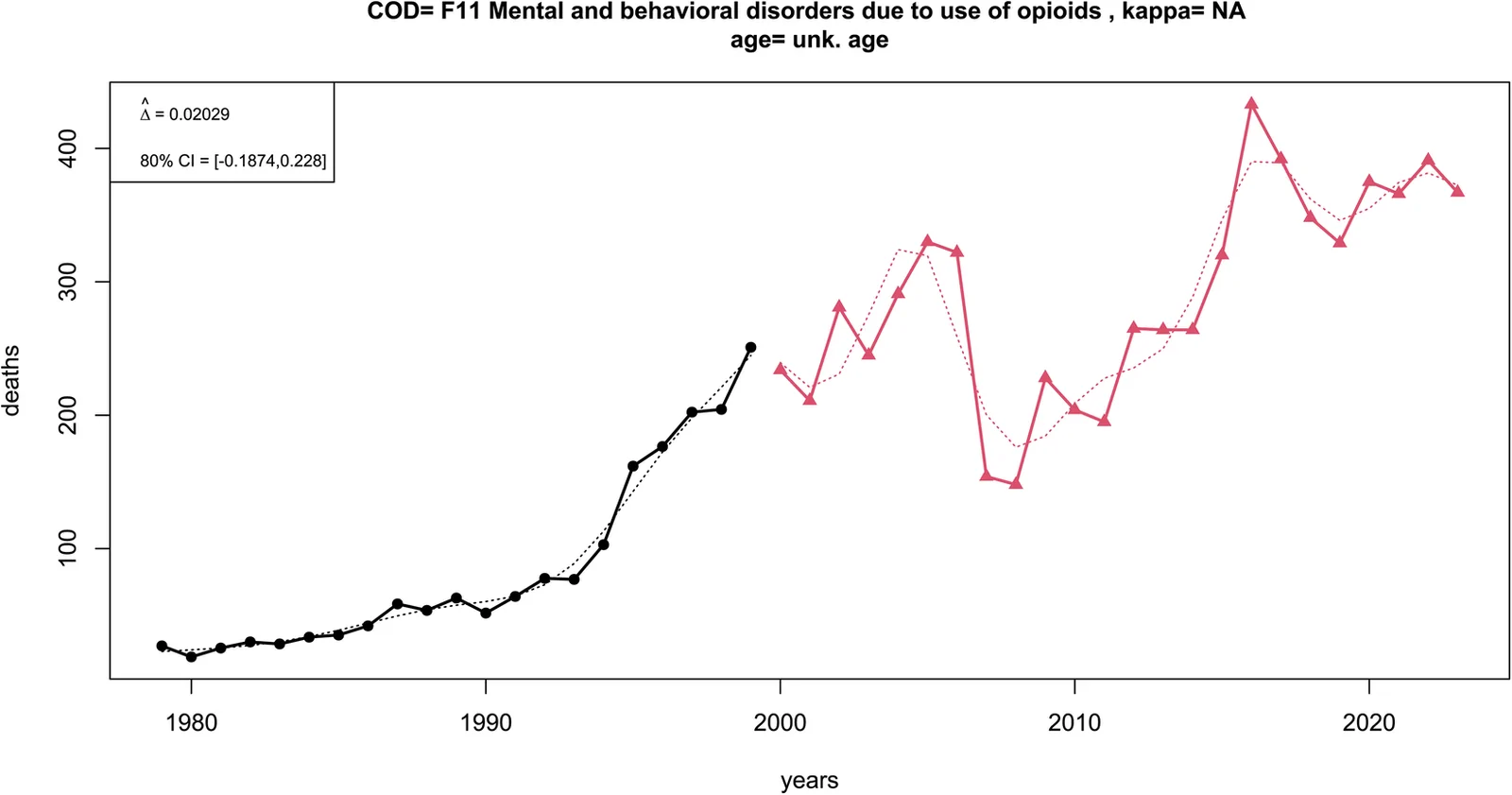
An example of a verification plot to check for remaining disruptions after reconstruction of the data series into ICD-10. Deaths from mental and behavioral disorders due to opioid use (ICD-10 code F11) in the United States are represented by the black line for the ICD-9 period (1979-1997) and the red line for the ICD-10 period (1998-2023) after reclassification of deaths for 1979-1997 into ICD-10 codes. The non-parametric P-sline smoothing for each period is depicted by the dotted lines (black for the period from 1979 to 1997, red for the period from 1998 to 2023). The 80% confidence interval of the difference ($\hat{\Delta }$) between the two smoothed series for the year of the transition from ICD-9 to ICD-10 (1998) includes zero, thus indicating that the reconstructed series does not exhibit a statistically significant disruption.

**Fig. 4:**
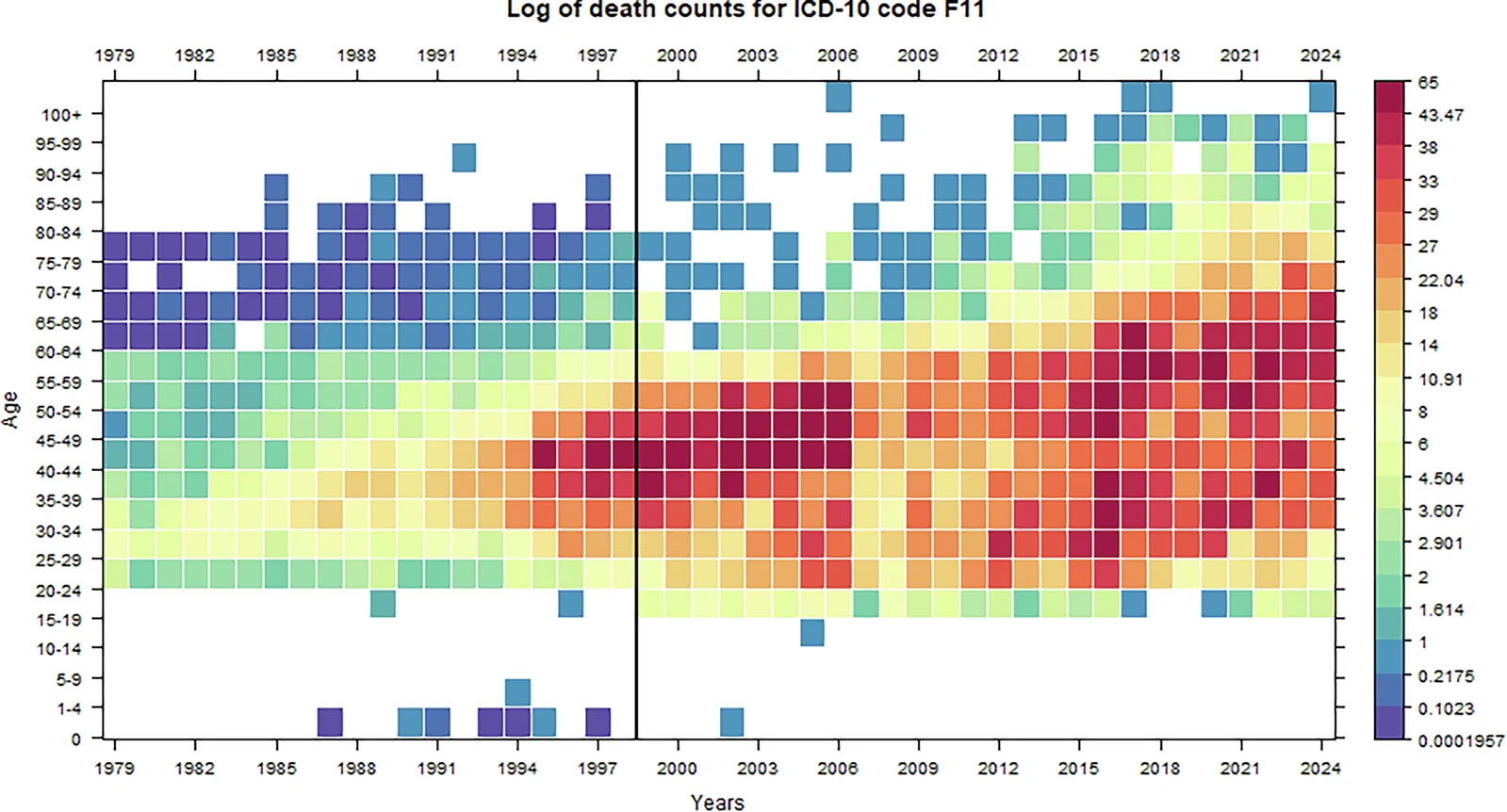
Example of a diagnostic level plot. The logarithms of the death counts from mental and behavioural disorders due to opioid use (ICD-10 code F11) in the United States are ploted for each five-year age group and each calendar year from 1979 to 2023. The number of deaths is indicated by the color (lowest in blue, highest in red). Though the total death counts has increased progressively over time, especially for working age adults, changes in the pattern do not show an obvious break at the time of the transition from ICD-9 to ICD-10 (in 1998), thus suggesting that the reconstruction is satisfactory.

A series of diagnostic charts (graphs and surfaces) are produced to identify any unexpected disruptions in the series, verify the plausibility of trends, detect possible outliers, and evaluate coherence of age patterns. Examples of two of these diagnostic charts are presented in Figs. 3, 4.

Figure 3 illustrates the method implemented to check for any remaining disruptions associated with an ICD change for a specific cause after all deaths have been reclassified into ICD-10. The method to determine whether a statistically significant disruption is associated with a change in the ICD was developed by Camarda and Pechholdová for this project^22^. Its goal is to detect discontinuities in a series of death counts for a particular cause (or ICD code) by means of forecasting the estimated trends, before and after changes in the ICD. The estimation of the trends is carried out by implementing non-parametric P-spline smoothing techniques on the standardized deaths within a Poisson framework, thus accounting for the variability in historical trends. The idea is to extrapolate the forecasted trends (forward for the death count series before the ICD change, i.e., for years 1979–1997 on Fig. 3, and backward for the death count series after the ICD change, i.e., for years 1998–2023) and check whether the difference in the two independently forecasted series at the mid-point (i.e., between 1997 and 1998 on Fig. 3) is statistically significantly different from zero. If it is, then a clear disruption is presumed at the time of the ICD-change. Statistically significant disruptions are evaluated in their specific medical and statistical contexts to determine whether they call for further adjustments, in which case additional work is performed to redistribute deaths from one ICD scheme into the next one. The illustration presented here relies on the reconstructed series of death counts for the United States over the period from 1979 to 2023 for ICD-10 code F11, which corresponds to mental and behavioral disorders due to opioid use. In the United States, the transition from ICD-9 to ICD-10 took place in 1998.

Figure 4 shows a surface plot (or heat map) of the age-specific death counts for the same cause as used on Fig. 3 (Mental and behavioral disorders due to opioid use – ICD-10 code F11) also for the United States. In this example, the age pattern of mortality from ICD-10 code F11 is as expected: some deaths of young children, who have presumably accidentally consumed the drug; a large and growing number of deaths for young adults over the period; and an apparent cohort effect. This type of plot is produced for every 3-digit ICD-10 code after deaths have been redistributed and all suspicious cases are carefully reviewed by the country specialist and the database administrators.

As in the HMD, relationships with staff at the National Statistics Offices in each country have been cultivated by the HCD country specialists over the years so that each anomaly in the data can be properly discussed with the official data providers. All unresolved issues are carefully documented in the country-specific documentation.

#### Sensitivity to methods

Each country is processed by a country specialist. The country specialists have been selected for their technical skills (most were trained as demographers, a few as statisticians) and experience with the country data. A standardized process has been established by the project team so that the reconstruction is carried out in a similar manner for all ICD transitions and across all countries. To reduce subjectivity as well as to save time, automated or semiautomated algorithms have been developed, carefully tested, and implemented at each step of the reconstruction process: the production of correspondence tables, the construction and visualization of the fundamental associations of items, the calculation of transition coefficients, and the identification and measurement of statistical disruptions and inconsistencies in the data series. The automation deals primarily with the implementation of ICD-10 (the transition from ICD-9 to ICD-10), as it represents the most challenging change to mortality statistics in the second half of the 20^th^ century (after the transition from ICD-5 to ICD-6) and enables reliance on the WHO ICD-9/ICD-10 Translator, which is based on hypothetical medical correspondences for most of the ICD-10 codes, and the results of bridge-coding studies in a number of countries^14^. In addition to minimizing manual effort, automation reduces the risk of mistakes and increases standardization of the process from one country to another. Statistical and graphical verifications are carried out by the HCD coordinator, the database administrators and the Directors to check that all steps have been followed properly and that the resulting data series are consistent over time.

The quality of the output series is predicated on the fundamental assumption that the redistribution coefficients estimated from the last year of one ICD period to the first year of the following ICD period are valid for the whole period when the prior ICD was implemented. There is no way of assessing the validity of this assumption with real data in the absence of continuous dual coding over an entire ICD period. Indeed, bridge-coding information, considered the gold standard to redistribute deaths from one classification scheme to another, cannot resolve this problem since bridge-coding studies have only been only implemented for a single calendar year for a given ICD transition in any given country. However, statistical consistency checks are implemented to verify the plausibility of this assumption in each of the HCD countries.

In addition, country- or time-specific variations in coding practices, data collection, and certification process may introduce heterogeneity into the data. However, the reconstruction method implemented in the project can be viewed as an *a posteriori* theoretical bridge-coding approach. Applied separately to each country, it helps smooth classification-induced discontinuities while accounting for country-specific patterns. Unlike conventional bridge-coding studies, which are often based on limited samples, this approach operates on the full set of available data and therefore avoids sampling-related bias. It also provides a consistent solution that does not require the substantial resources typically involved in bridge-coding operations. It is clear, however, that limitations remain in the reconstructed series and that the figures should not be taken at face value but as plausible estimates. We believe that in spite of these limitations, the Human Cause-of-death Data are helpful to monitor changes in the cause-of-death structures of low mortality populations, to identify drivers of the on-going longevity revolution, and to foster cross-country comparisons of modern mortality patterns.

#### Future work

In addition to periodic updates of the existing data series in the HCD as new data is produced by National Statistics Offices, typically annually, we are planning to extend our reconstruction methods to new countries and to additional historical periods wherever possible, at least back to the beginning of the ICD-6 period, given the difficulties to reconcile the 5^th^ and 6^th^ Revisions of the ICD as previously mentioned. One of the advantages of the HCD methods is their flexibility: not only can the methods be adapted to countries which had adopted schemes of disease classifications different from the standard promoted by the World Health Organization (e.g., the Soviet classifications) but we will also be able to implement it when and where ICD-11, the new Revision of the International Classification of Diseases, is implemented. In countries which will carry out bridge-coding studies for the transition from ICD-10 to ICD-11, we will make use of the results to improve accuracy as we have done in the past, supplementing official bridge-coding studies with our own methods where necessary (for instance where the bridge-coding will have been conducted on a sample of deaths rather than on all deaths for a given year).

## Usage Notes

This paper presents a dataset with cause-specific mortality series (death counts, crude death rates, age-specific and age-standardized death rates) harmonized into the 10^th^ Revision of the International Classification of Diseases over extended and varying time periods for 18 countries with high quality data. A snapshot of the data with all statistical series and all associated documentation bundled into a single compressed file is provided at the Harvard Dataverse^28^. When the file is uncompressed, the data are distributed into one directory for each country (named for their ISO-3 country code) and a separate directory titled “docs” for the metadata (with the long, intermediate, and short lists of cause-of-death categories, the HCD citation list, a description of the methods implemented and the format of the data series). Three types of data files are available: the series reconstructed into ICD-10 (in the “Reconstructed” directory), the data in their original classifications where we are authorized to provide access to the raw data (in the “OriginalClassification” directory), and the birth and population files used to compute all mortality statistics. In addition, a country-specific documentation is provided in a sub-directory within each country directory that includes the full list of cause-of-death categories for that country, a reference file with a detailed description of all sources for the original data and the information needed to retrieve them, and a background and documentation file presenting a historical account of cause-of-death data collection systems, the timing of implementation of each Revision of the ICD, a discussion of data idiosyncrasies and limitations and, where applicable, the particular adjustments that have been carried out on the raw data outside of the standard HCD process.

Periodically updated data series are available with version control on the database website^27^. The database website provides access to a compressed file with data for all countries and all statistics as well as with all associated documentation that is exactly similar in structure and format to the snapshot accessible on the Harvard Dataverse^28^, but it also allows users to download data for a single country and/or a single mortality statistic at a time. The data are accessible to everyone for free upon a short registration process implemented to help us keep track of usage. For users interested in a particular country, the country-specific pages provide access to four different blocks of data: (1) the reconstructed mortality series, that take changes in the ICD into account and after redistribution of deaths from ill-defined and unknown causes; (2) the death tabulations for ill-defined or unknown causes; (3) the mortality series in their original classification, that is with deaths classified into the ICD in operation at the time they occurred, before redistribution of deaths from ill-defined and unknown causes (only for those countries which authorized publication of the raw aggregated data – Fig. 1a); and (4) the birth and population data used for the calculation of the mortality rates.

For any additional information regarding the raw data or for any other questions about the HCD, users are encouraged to reach out to the program team at the generic address for all HMD-related matters (hmd@mortality.org).

## Data Availability

All the HCD data presented in this article are freely available from the HMD website (www.mortality.org/Data/HCD) to use with appropriate citations under the Creative Commons Attribution 4.0 International Public License. In addition, a snapshot of the data available at the time of this writing is provided in the Harvard Dataverse at [https://dataverse.harvard.edu/dataverse/HCD/_data/_paper] from [https://doi.org/10.7910/DVN/L43DMH]. With some exceptions, as further explained below, the raw data used to create the HCD data series are protected and cannot be distributed due to data privacy laws.

## Code availability

All R scripts^31^ used to construct and verify the published data series after the HCD country specialists have provided the input files have been packaged and made available on the Harvard Dataverse website^32^. There are two distinct sets of scripts, depending on whether a country has only implemented the successive ICDs or whether it has, over any periods of time, implemented a Soviet-related classification. We have posted raw data for two example countries together with these two sets of scripts on the Harvard Dataverse website. The two illustrative countries are the United States, as an example of a country that has only implemented the standard ICDs, and Ukraine, as an example of a country that has implemented a Soviet-inspired classification before using the ICD. Instructions to implement the computer codes are provided on the Harvard Dataverse alongside the codes.
